# Therapeutic Rationale to Target Highly Expressed Aurora kinase A Conferring Poor Prognosis in Cholangiocarcinoma

**DOI:** 10.7150/jca.31989

**Published:** 2020-02-03

**Authors:** Xiwei Ding, Tianlu Huang, Chunyan Peng, Keun Soo Ahn, Jesper B Andersen, Monika Lewinska, Yu Cao, Guifang Xu, Gang Chen, Bo Kong, Helmut Friess, Shanshan Shen, Lewis R Roberts, Lei Wang, Xiaoping Zou

**Affiliations:** 1 Department of Gastroenterology, Nanjing Drum Tower Hospital, The Affiliated Hospital of Nanjing University Medical School, Nanjing, Jiangsu, China; 2 Division of Hepatobiliary and Pancreatic Surgery, Department of Surgery, School of Medicine, Keimyung University Dong San Medical Center, Daegu, The republic of Korea; 3 Biotech Research and Innovation Centre, Department of Health and Medical Sciences, University of Copenhagen, Copenhagen, Denmark; 4 Division of Hepatobiliary Surgery, The First Affiliated Hospital of Wenzhou Medical University, Wenzhou, Zhejiang, China; 5 Department of Surgery, Technical University of Munich, Munich, Germany; 6 Division of Gastroenterology and Hepatology, College of Medicine, Mayo Clinic and Mayo Clinic Cancer Center, Rochester, MN, US

**Keywords:** Aurora kinase A, Alisertib, Cholangiocarcinoma, mitotic spindle checkpoint, poor prognosis, molecular marker

## Abstract

**Background**: Cholangiocarcinoma is a highly lethal neoplasm for which the currently available chemotherapeutic agents are suboptimal. Numerous studies show that alterations in expression of genes related to mitotic spindle and mitotic checkpoint are involved in chromosomal instability and tumor progression in various malignancies. This study aimed to evaluate these genes in cholangiocarcinoma patients.

**Material and methods**: Different public datasets were analyzed to examine the expression of 76 selected mitotic spindle checkpoint genes including Aurora Kinase A (AURKA) in cholangiocarcinoma. Afterwards, cell number counting, CCK-8 assay, and Caspase 3/7 assay were used to explore the antitumor effect of AURKA inhibitor Alisertib *in vitro*. In addition, xenograft model was used to evaluate the antitumor effect of Alisertib *in vivo*. Furthermore, siRNA mediated silencing of AURKA was used to verify the function of AURKA in cholangiocarcinoma.

**Results**: Components of the mitotic spindle checkpoint, including AURKA, were broadly dysregulated in human cholangiocarcinoma. High AURKA mRNA expression was associated with poor survival in cholangiocarcinoma patients within different datasets. AURKA specific inhibitor Alisertib, inhibited cell growth, induced cell cycle arrest in G2/M phase, and promoted apoptosis in cholangiocarcinoma cell lines. Additionally, Alisertib also inhibited tumor growth in a cholangiocarcinoma xenograft mouse model. Furthermore, AURKA knockdown by siRNA recapitulated the antitumor effect of Alisertib. AURKA expression was also highly correlated with its interaction proteins Polo-like kinase 1(PLK1) and Targeting protein for xenopus kinesin-like protein2 (TPX2) in different cholangiocarcinoma datasets.

**Conclusions**: Highly expressed AURKA confers poor outcomes in cholangiocarcinoma and may represent a rational therapeutic target.

## Introduction

Cholangiocarcinoma is a highly malignant tumor with dismal prognosis. Despite recent advances in diagnosis and treatment, 5-year survival rate of cholangiocarcinoma does not increase significantly and remains at 10% 1. Gemcitabine-based chemotherapies are recommended for patients with inoperable cholangiocarcinoma 2. However, long term outcome is still poor, highlighting the need for the identification of novel therapeutics for this neoplasm.

Many studies show that alterations in expression of genes related to mitotic spindle and mitotic checkpoint are involved in chromosomal instability and tumor progression in various solid and hematologic malignancies 3-5. However, despite the importance of the mitotic spindle checkpoint in cancer, detailed analyses of mitotic spindle checkpoint gene expression in cholangiocarcinoma has not yet been performed.

Mammalian Aurora family of serine/threonine kinases are key regulators of mitotic progression and cell proliferation. Among the three members of the Aurora kinase family (Aurora kinase A, B, and C), Aurora kinase A (AURKA) and Aurora kinase B (AURKB) are expressed at detectable levels in somatic cells undergoing mitotic cell division. Aberrant Aurora kinase A activity has been implicated in oncogenic transformation through the development of chromosomal instability and tumor cell heterogeneity 6, 7. Recent studies also reveal a novel non-mitotic role of Aurora kinase A activity in promoting tumor progression through regulating a number of key oncogenic signaling pathways 8, 9. Therefore, AURKA represents an attractive target for cancer therapeutics.

In the present study, we examined the expression of 76 selected mitotic spindle checkpoint genes known to be involved in various molecular mechanisms associated with the mitotic spindle checkpoint in cholangiocarcinoma 3. Components of the mitotic spindle checkpoint, including AURKA, were broadly dysregulated in human cholangiocarcinoma patients using The Cancer Genome Atlas (TCGA) or Gene Expression Omnibus (GEO) datasets. We also found that highly expressed AURKA was a negative prognostic marker of cholangiocarcinoma. We hypothesized that this can be exploited therapeutically with AURKA inhibition, and we tested the antitumor activity of the selective AURKA inhibitor Alisertib* in vitro* in cholangiocarcinoma cell lines and *in vivo* in a mouse xenograft model.

## Materials and Methods

### Reagents

Alisertib was purchased from Selleck (Houston, TX, USA). Anti-β-actin primary antibody was purchased from Sigma Aldrich (St. Louis, MO, USA). Antibody against human AURKA (#14475) was purchased from Cell Signaling Technology (Beverly, MA, USA). Immobilon Western Chemiluminescent HRP detection kit was from Millipore (Burlington, MA, USA). Cell counting kit-8 (CCK-8) was from Dojindo Laboratories (Kyushu, Japan). Caspase-Glo 3/7 assay kit was from Promega (Madison, WI, USA). Annexin V-FITC Apoptosis Detection kit was from BD Pharmingen (Franklin Lakes, NJ, USA). Lipofectamine RNAiMAX was from Invitrogen (Carlsbad, CA, USA). Cell culture medium was obtained from Gibco (Grand Island, NY, USA). Fetal bovine serum (FBS) was from Biological Industries (Kibbutz Beit Haemek, Israel). Cell extraction buffer was from Life Technologies (Grand Island, NY, USA). Alisertib was dissolved in DMSO to make a stock solution of 10 mM.

### The Cancer Genome Atlas (TCGA) and public microarray data analysis

TCGA cholangiocarcinoma transcriptomic dataset consisting of 36 cholangiocarcinoma patients and 9 normal bile ducts was downloaded from the Firehose run of the Broad Genome Data Analysis Center on May 6, 2017 (http://gdac.broadinstitute.org). The TCGA data consists of 36 cholangiocarcinoma samples and 9 normal bile duct tissue samples. Cholangiocarcinoma transcriptomic microarray dataset GSE107943 was built by the co-author 10, which consists of 30 intrahepatic cholangiocarcinoma surgical specimens and 28 non-cancerous surrounding liver specimens. In the dataset GSE107943, 2 molecular subtypes of iCCA with distinct clinicopathological differences were identified. Another public cholangiocarcinoma microarray profiling dataset GSE26566 was downloaded from the Gene Expression Omnibus (GEO). GSE26566 was built by Andersen et al from Copenhagen 11, which consists of 104 cholangiocarcinoma samples, 59 non-cancerous surrounding liver samples, and 6 normal bile duct samples. Through analyzing the dataset GSE26566, the author identified 2 prognostic categories of patients with CCA, each containing 2 subclasses characterized by distinct gene expression profiles. Additionally, cholangiocarcinoma dataset (EGAD00001001693) constructed by Nakamura et al from Japan and stored in European Genome-phenome Archive database was analyzed to explore the association of AURKA mRNA expression with survival 12.

### Cell culture

Five human cholangiocarcinoma cell lines HCCC9810, HuCCT1, RBE, HuH28, and OZ were used. HuH28, OZ, and HuCCT1 were provided by Lewis R.Roberts (Mayo Clinic, MN, USA), which were originally obtained from the Japanese Collection of Research Bioresources. RBE and HCCC9810 were obtained from Cell Bank of Chinese Academy of Sciences (Shanghai, China). Cell lines were authenticated using short tandem repeat profiling. All cholangiocarcinoma cell lines used were cultured in RPMI 1640 with 10% FBS and maintained at 37°C in the presence of 5% CO_2_.

### Cell proliferation and viability assay

Cells were plated in 6-well plates at 1×10^5^ cells/well. After 24 h, drugs were added and cells were incubated for the indicated time. Cell proliferation was detected by cell number counting with trypan blue. Cell viability was detected by CCK-8 assay. Cells were seeded into 96-well plates at 3000 cells/well in triplicate, cultured overnight then treated with drugs for indicated time. The CCK-8 assay was performed as previously described 13.

### Colony formation assay

Cells were plated at 500 cells/well in a 6-well plate. After 24 h, drugs were added and cells were incubated for 7 days. Cells were then fixed with methanol solution and stained with 0.5% crystal violet. The number of colonies, defined as ≥ 50 cells/ colony, was counted manually by light microscopy.

### Cell Cycle analysis

Cells were seeded in 6-well plates at 2×10^5^ cells/well and treated with varying concentrations of Alisertib or transfected with siRNA targeting AURKA for 48-72 h.

Cell cycle was analyzed using BD Cycletest Plus DNA Reagent Kit Cells according to the manufacturer's instructions. Cell cycles were analyzed by using FlowJo software.

### Annexin V-FITC apoptosis assay

Cells were seeded in 6-well plates at 2×10^5^ cells/well and treated with varying concentrations of Alisertib or transfected with siRNA targeting AURKA for 48-72 h. Apoptosis was assessed using the Annexin V-FITC Apoptosis Detection kit and performed according to the manufacturer's instructtions. Data were analyzed using FlowJo software.

### Caspase 3/7 activity assay

Caspase 3/7 activity was analyzed using the Caspase-Glo 3/7 assay kit according to the manufacturer's instructions. 3000 cells were seeded into 96-well white opaque plates and a corresponding optically clear 96-well plate, and then allowed to adhere overnight. The next day, cells were treated with varying concentrations of indicated drugs for 48 h. At the end of the incubation time, Caspase-Glo reagent was added to each well. Plates were gently mixed and incubated for 1 h at room temperature. The luminescence was then measured in a GloMax Luminometer (Promega, Madison, WI). The corresponding 96-well clear plate was used to measure the relative number of viable cells with the CCK-8 assay. Caspase 3/7 activity was normalized to viable cell number.

### Western immunoblotting

Whole-cell lysates were prepared in Cell extraction buffer, and western immunoblotting analysis was performed as previously described 13. Twenty micrograms of protein were separated on a 4-20% Tris-HCl gel and transferred to PVDF membranes. Membranes were probed with the appropriate primary antibodies. Blots were then incubated with horseradish peroxidase-conjugated secondary antibodies and signals were visualized using the HRP detection kit. β-actin was used as a loading control.

### RNA interfering

Cells were grown in 6-well plates and transfected with 20 nM siRNA using Lipofectamine RNAiMAX (Life Technologies) according to the manufacturer's protocol. The siRNA AURKA (5'- GAAGAGAGTTATTCATAGA-3') and siRNA control duplexes were purchased from RiboBio (RiboBio Co. Ltd., Guangzhou, China).

### Xenograft in nude mice

Nude mice were purchased from Beijing Vital River Laboratory Animal Technology. The animal experiments were approved by the Institutional Animal Care and Use Committee. HuCCT1 cells (3 × 10^6^ cells) were suspended in 100 μl serum free medium, mixed with 100 μl Matrigel, and injected subcutaneously into the right flank of 4- to 6-week old female nude mice. After 10 days when the tumor size reached about 150-200 mm^3^, mice were randomized into two groups with 6 mice in each group. Mice were treated by 30 mg/kg of Alisertib (daily × 5 days per week, by oral gavage) or vehicle control (10% 2-hydroxypropyl-b-cyclodextrin and 1% sodium bicarbonate, daily × 5 days per week, by oral gavage). Tumor volume and animal weight were measured twice a week. Tumor size was measured with digital caliper and calculated as V = L×S^2^/2 (where L is the longest diameter and S is the shortest diameter).

### Statistics

Statistical tests were conducted with GraphPad Prism 6.0. The IC_50_ was calculated using nonlinear regression analysis in Prism 6.0. For comparisons between two groups, parametric Student's t test or nonparametric Mann-Whitney test were used. In experiments involving more than two groups, one-way ANOVA with a Turkey post hoc test was used. Kaplan-Meier survival analysis was used to estimate the survival distributions. The log-rank test was used to assess the statistical significance between the stratified survival groups. The correlations of gene expression were analyzed using Pearson correlation. Results were considered statistically significant at* P*<0.05.

## Results

### Mitotic spindle checkpoint genes were overexpressed in cholangiocarcinoma

To identify the differentially expressed mitotic spindle checkpoint genes in cholangiocarcinoma tissues, we analyzed the TCGA cholangiocarcinoma and normal bile duct tissue RNA sequencing data and two microarray datasets (GSE107943 and GSE26566).

**Figure 1:**
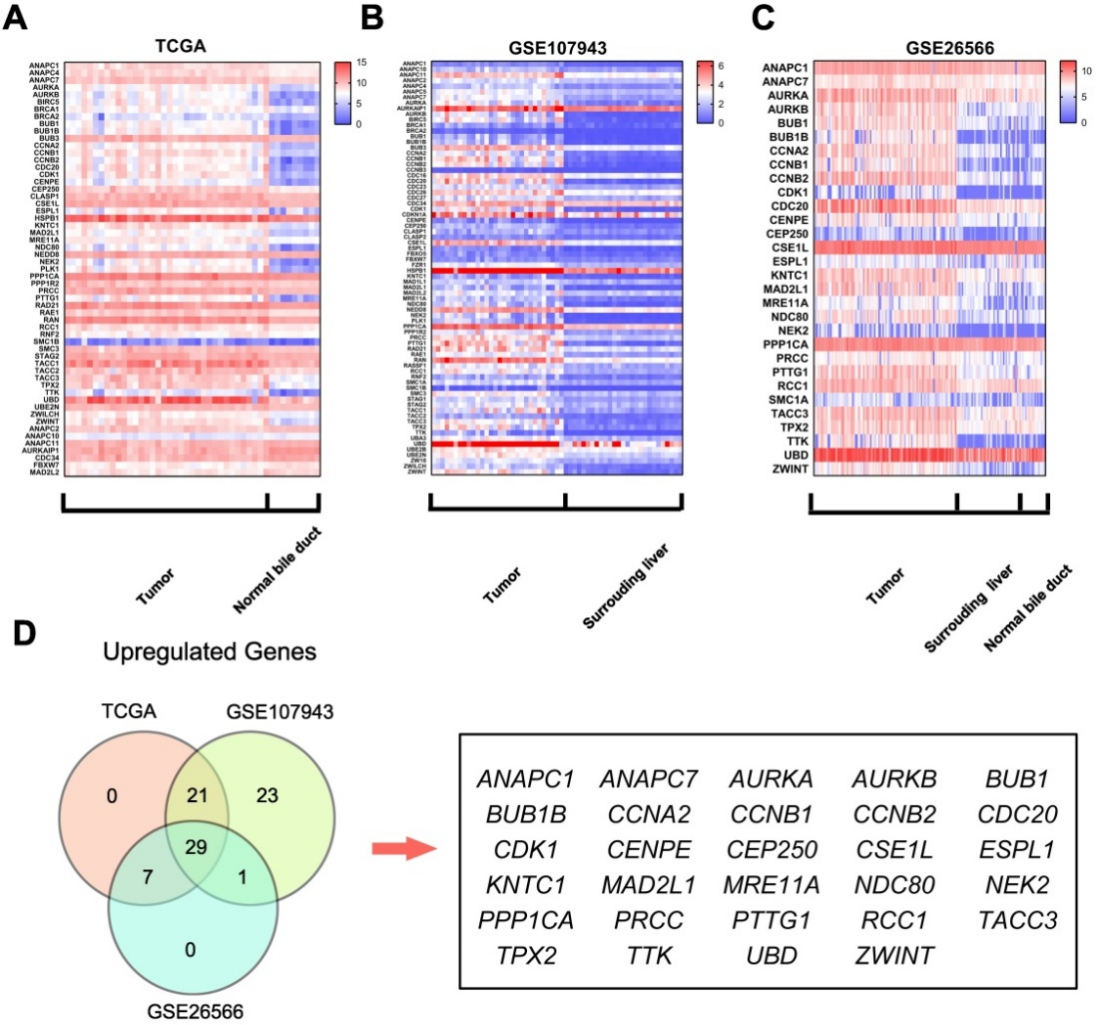
** Mitotic spindle checkpoint genes were broadly overexpressed in human cholangiocarcinoma. A)** A heat map was drawn to show the differentially expressed gene transcripts related to the mitotic spindle checkpoint in cholangiocarcinoma tissues and normal bile duct tissues in the TCGA RNA sequencing data. **B-C)** Heat maps were drawn to show the differentially expressed gene transcripts related to the mitotic spindle checkpoint in cholangiocarcinoma tissues, normal surrounding liver tissues and normal bile duct tissues in the GSE107943 and GSE26566 datasets. **D)** Venn diagram of dysregulated genes in TCGA, GSE107943, and GSE26566 datasets.

Further analyses of these data showed that 57 genes were differentially expressed in the TCGA dataset (50 upregulated and 7 downregulated, Fig.1A); 74 were differentially expressed in the GSE107943 dataset (74 upregulated and 0 downregulated, Fig.1B); 30 were dysregulated in the GSE26566 dataset (30 upregulated and 0 downregulated, Fig.1C). Further Venn analyses revealed that 29 genes (38.2%) were consistently upregulated in three datasets (Fig.1D). These findings indicate that components of the mitotic spindle checkpoint were broadly dysregulated in human cholangiocarcinoma, and part of those genes may be useful biomarkers for cholangiocarcinoma diagnosis.

### AURKA expression was significantly upregulated in cholangiocarcinoma, correlated with cell proliferation, and predicted poor survival

**Figure 2:**
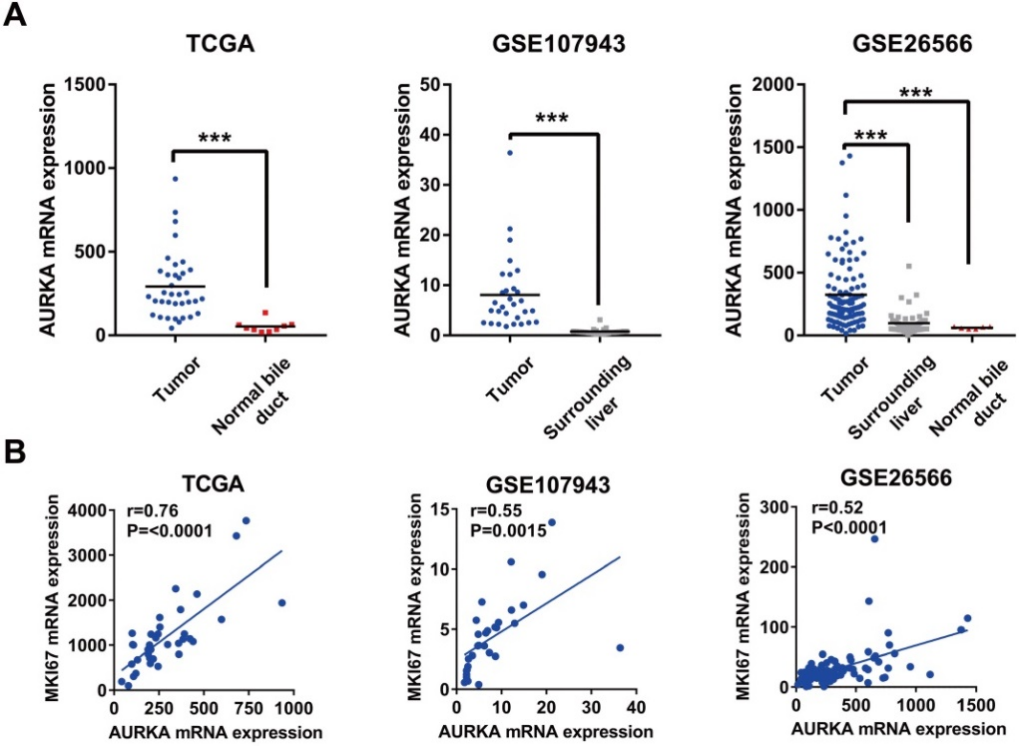
AURKA expression was upregulated in cholangiocarcinoma and correlated with cell proliferation. A) AURKA mRNA expression was markedly upregulated in cholangiocarcinoma in TCGA, GSE107943, and GSE26566 datasets. **B)** The expression level of AURKA showed a significant positive correlation with Ki-67 expression in the dataset of TCGA, GSE107943, and GSE26566. The r and P values were determined by Pearson correlation analysis.

**Figure 3:**
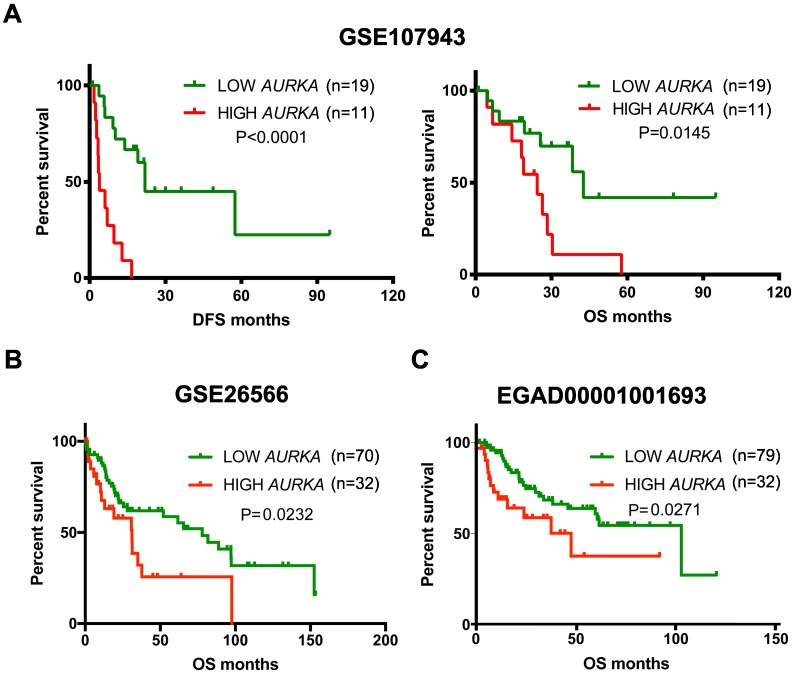
** High AURKA expression correlated with poor survival in cholangiocarcinoma tumors. A)** Kaplan-Meier survival curves showing the relationship between AUKRA mRNA expression and disease free survival and overall survival in the Korea cohort GSE107943 (n = 30). **B)** Kaplan-Meier survival curves showing the relationship between AURKA mRNA expression and overall survival in the Copenhagen cohort GSE26566 (n = 102). **C)** Kaplan-Meier survival curves showing the relationship between AURKA mRNA expression and overall survival in the Japan cohort EGAD00001001693 (n=111).

Given the oncogenic potential of AURKA in other cancers and a highly specific inhibitor of AURKA is already available, we focused our further descriptive and functional analyses on AURKA. As shown in Fig. 2A, AURKA mRNA was markedly higher in the cholangiocarcinoma compared to that in the normal bile ducts or surrounding normal livers in all three datasets. Next, analyses using data of TCGA, GSE26566, and GSE107943 revealed mRNA expression levels of AURKA positively correlated with that of the proliferative marker Ki-67 (Fig. 2B). Furthermore, we analyzed the association of AURKA mRNA expression with cholangiocarcinoma outcomes in different cohorts using the mean AURKA expression value as a cut-off. In the South Korea Cohort (GSE107943), Kaplan-Meier survival demonstrated that high AURKA mRNA expression was strongly correlated with reduced disease free survival (DFS) time (P<0.0001) and overall survival (OS) time (P=0.0145) (Fig. 3A). In the Copenhagen cohort (GSE26566), AURKA mRNA expression also correlated with cholangiocarcinoma overall survival time. Patients with higher AURKA mRNA levels in cholangiocarcinoma tumors showed significantly shorter overall survival time than those with lower levels of AURKA (P=0.0232) (Fig. 3B). Further analysis was carried out using a publicly available transcriptomic dataset (Japan Cohort), which also showed high AURKA mRNA expression was significantly correlated with reduced overall survival time (P=0.0271) (Fig. 3C). Collectively, these data indicate that highly expressed AURKA may be a negative prognostic factor in cholangiocarcinoma.

### AURKA specific inhibitor Alisertib inhibited cell proliferation of cholangiocarcinoma cells *in vitro*

**Figure 4:**
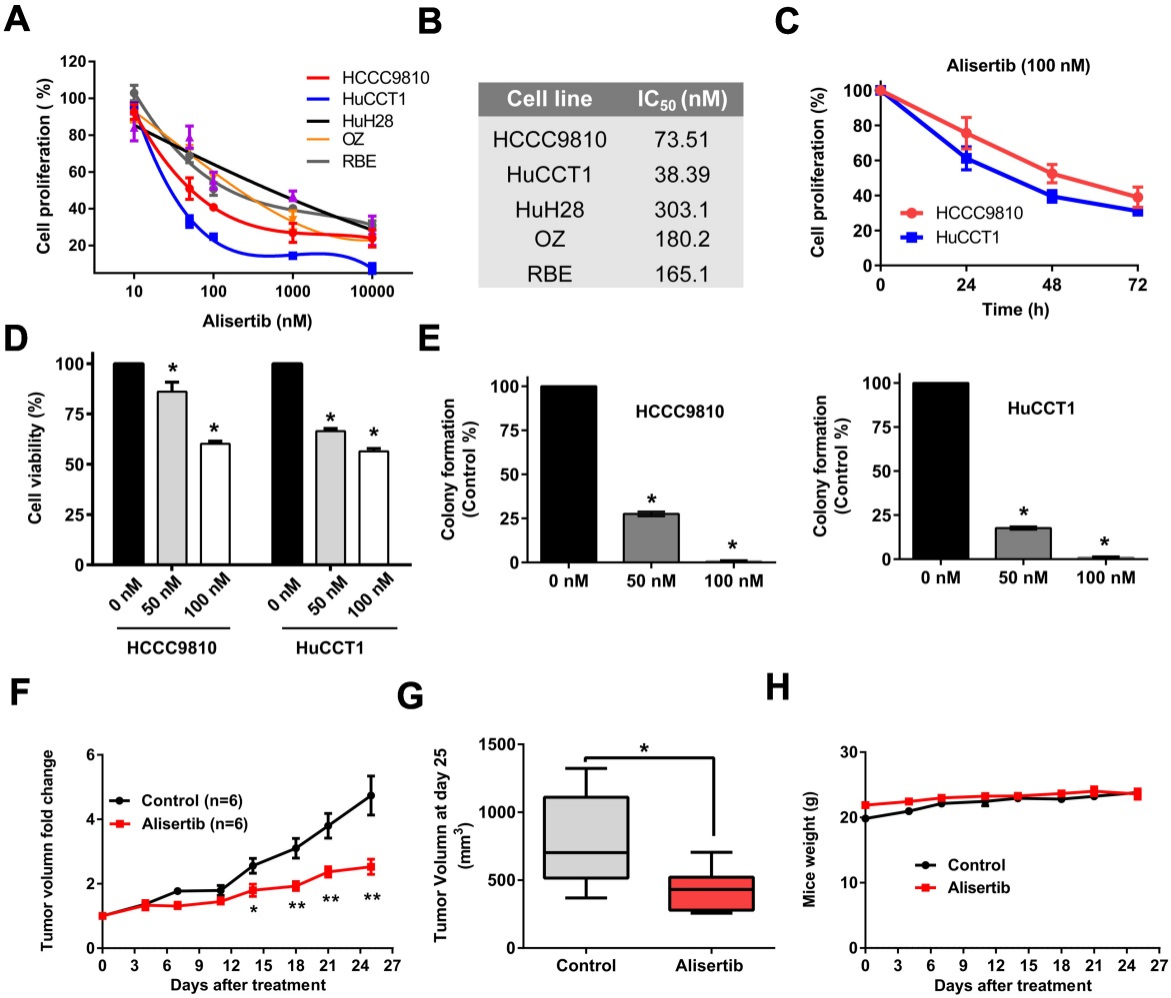
** Effect of Alisertib on tumor growth *in vitro* and *in vivo*. A)** Cells were treated with Alisertib at different concentrations (10nM-10 μM) for 72 h and cell proliferation was determined by cell number counting. **B)** IC_50_ values of Alisertib in cholangiocarcinoma cells following 72 h of treatment. **C)** Time-response curves of HCCC9810 and HuCCT1 cell lines to Alisertib treatment (100 nM). **D)** HCCC9810 and HuCCT1 were treated with Alisertib at different concentrations for 72 h and cell viability was determined by CCK-8 assay. **E)** Plated HCCC9810 and HuCCT1 were treated with Alisertib for 7 days and colonies were stained with 0.5% crystal violet. **F)** HuCCT1 cells were injected subcutaneously into the right flanks of athymic nude mice. When tumors reached a size of approximately150- 200 mm^3^, mice were randomized to receive vehicle (n=6) or Alisertib (n=6) by oral gavage. Tumor volume was measured every 3-4 days. Values shown are the mean difference in tumor volume ± SEM. **G)** Tumor volume in each group on day 25. Data represented are mean ± min/max group values. **H)** Change of body weight in mice treated with vehicle control or Alisertib. All results are presented as mean ± SEM from 3 independent experiments unless otherwise pointed, * P < 0.05, ** P < 0.01, compared with control.

To determine whether AURKA may represent an appropriate therapeutic target in cholangiocarcinoma, we initially screened a panel of cholangiocarcinoma cell lines with Alisertib, a highly selective AURKA inhibitor. Cholangiocarcinoma cell lines exhibited different sensitivity to AURKA inhibition (Fig. 4A) with IC_50_ values ranging from 38.4 nM to 303.1 nM (Fig. 4B). We compared their IC_50_ *in vitro* with their Cmax (maximum concentration) in blood using previous available preclinical pharmacokinetic data and phase I human clinical trial 14, 15. We found their IC_50_s were achievable *in vivo*. Because HCCC9810 and HuCCT1 cell lines were most sensitive to Alisertib, we further assessed the antitumor effect of Alisertib in these two cell lines. As shown in Fig. 4C, 100 nM Alisertib time-dependently inhibited cell proliferation of HCCC9810 and HuCCT1. CCK-8 viability assay and colony formation assay also verified the tumor inhibitory effect of Alisertib in these two cell lines (Fig. 4D and 4E).

### Alisertib inhibited cholangiocarcinoma growth in a xenograft mouse model

We next determined the effect of Alisertib on the growth of cholangiocarcinoma xenografts *in vivo*. To test this, we utilized the HuCCT1 cell line which exhibits sensitivity to Alisertib *in vitro*, and which has a tumorigenicity *in vivo*. HuCCT1 cells were engrafted into the right flank of nude mice. Ten days after implantation when tumors reached a size of approximately 150- 200 mm^3^, mice were randomly divided into 2 groups. Alisertib treatment induced a statistically significant inhibition of tumor growth when assessed by relative tumor volume change at days 14, 18, 21 and 25 (Fig. 4F) and when assessed by tumor volume at the completion of the experiment on day 25 (Fig. 4G and Supplemental Fig. 1). Notably, Alisertib did not affect mice weight (Fig.4H).

### Alisertib induced G2/M phase arrest and apoptosis of cholangiocarcinoma cells

**Figure 5:**
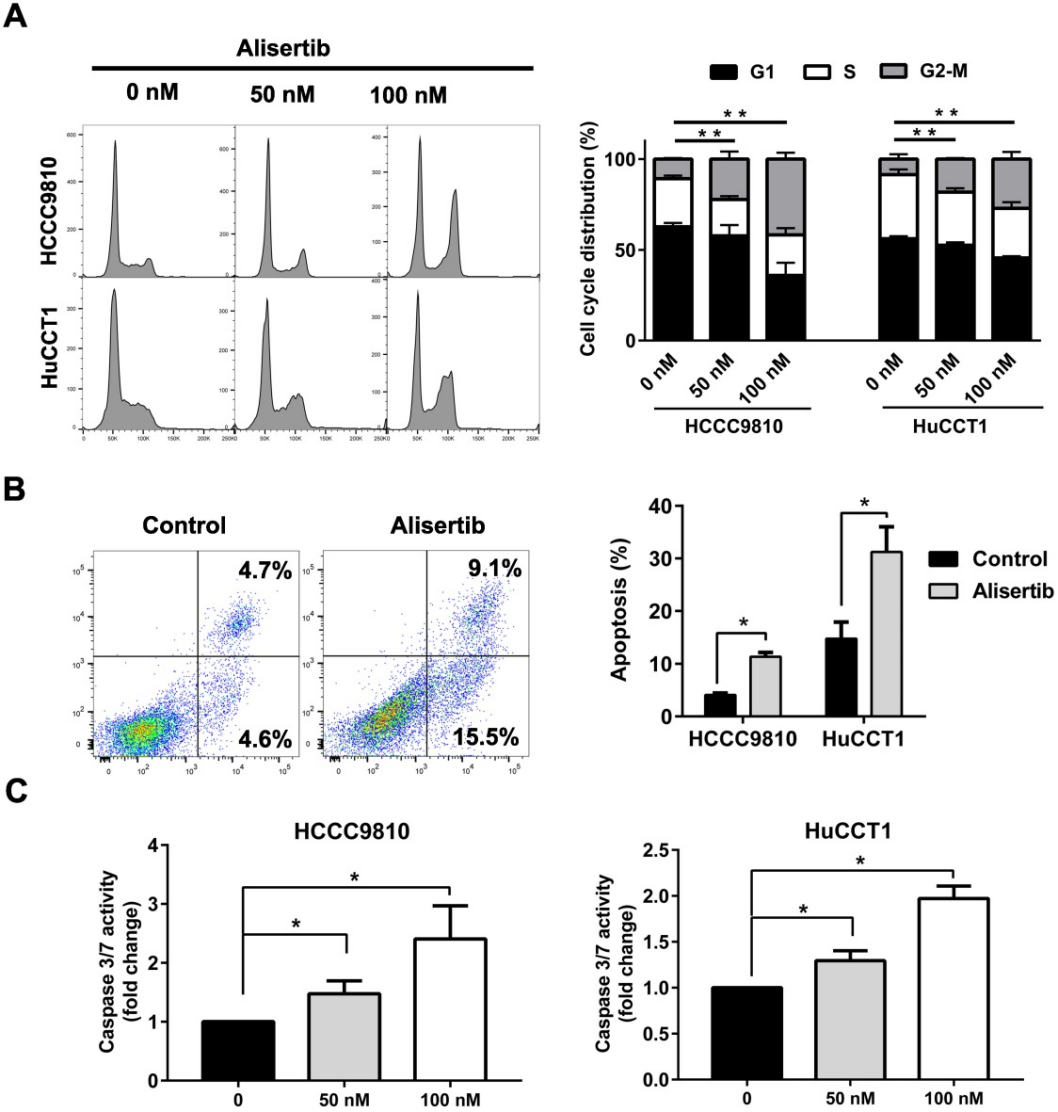
** AURKA inhibition induced cell cycle arrest and apoptosis. A)** Quantification of percentage of HCCC9810 and HuCCT1 cells in different cell cycle phases following treatment with Alisertib for 48 h. **B)** Apoptosis analysis via Annexin V/propidium iodide staining of cholangiocarcinoma cells at 100 nM Alisertib following 72 h of treatment. **C)** Apoptosis analysis via Caspase 3/7 activity of cholangiocarcinoma cells at various concentrations of Alisertib following 48 h of treatment. All data shown were Mean ± SEM from 3 independent experiments, * P < 0.05, ** P < 0.01, compared with control.

Then, we aimed to explore the mechanism underlying the tumor-suppressing effect of Alisertib. Treatment with Alisertib for 48 h induced cell cycle arrest in G2/M phase in both HCCC9810 and HuCCT1 cells (Fig. 5A). Significant apoptosis were found after treatment of 100 nM Alisertib for 72 h in both cell lines (Fig. 5B). Furthermore, treatment with Alisertib for 48 h significantly increased Caspase 3/7 activity, suggesting Alisertib may induce caspase-dependent apoptosis in cholangiocarcinoma cells (Fig. 5C). Collectively, these data demonstrated that AURKA played a tumor promoting role in cholangiocarcinoma by regulating cell cycle and apoptosis.

### AURKA knockdown was sufficient to recapitulate the antitumor effect of Alisertib

**Figure 6:**
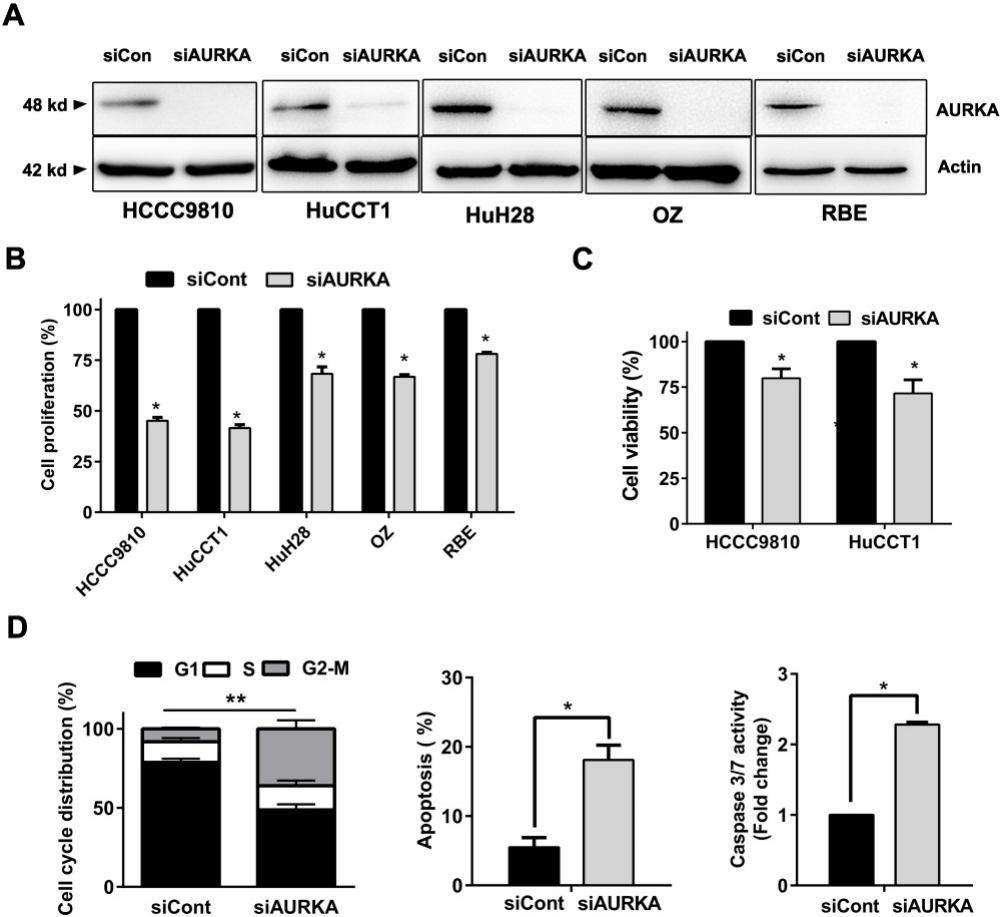
** Knockdown of AURKA diminished cell proliferation, viability, and increased G2/M phase arrest and apoptosis. A)** Western immunoblotting analysis of AURKA protein expression 48 h post-siRNA knockdown of AURKA in cholangiocarcinoma cells. **B)** Cell proliferation following 72 h AUKRA knockdown in cholangiocarcinoma cells. Cell proliferation was determined by cell number counting. Cells number was normalized to siControl (siCont). **C)** CCK-8 cell viability assay following 72 h AURKA knockdown in HCCC9810 and HuCCT1 cells (n= 3). Cell viability was normalized to siControl. **D)** Cell cycle analysis following 48 h AURKA knockdown in HCCC9810 cells. E) Annexin V-PI assay and Caspase 3/7 activity analysis of apoptosis following 72 h AURKA knockdown in HCCC9810 cells. All data shown were Mean ± SEM from 3 independent experiments, * P < 0.05, ** P < 0.01, compared with control.

Our results support that AURKA sustains proliferation in cholangiocarcinoma. We depleted AURKA to further elucidate Aurora kinase A is responsible for the cell proliferation of cholangiocarcinoma cells. SiRNA mediated suppression of AURKA were conducted in five cholangiocarcinoma cell lines and resulted in prominent reduction of protein expression at 48 hours post transfection (Fig. 6A). AURKA knockdown caused a significant reduction in cell proliferation (Fig. 6B). HCCC9810 and HuCCT1 cells were most sensitive to AURKA knockdown consistent with their response to AURKA inhibitor Alisertib. CCK-8 assay also showed that siRNA mediated knock down of AURKA inhibited cell viability in these cells (Fig. 6C). Furthermore, AUKRA silencing caused cell cycle arrest in G2/M phase, induced apoptosis and activated Caspase 3/7 activity in HCCC9810 cells (Fig. 6D). Taken together, our results showed that AURKA silencing by siRNA is sufficient to recapitulate the oncosuppressive effects of AURKA chemical inhibition on cholangiocarcinoma cells.

### AURKA interaction genes Targeting protein for xenopus kinesin-like protein 2 (TPX2) and Polo-like kinase 1 (PLK1) correlated with AURKA expression in cholangiocarcinoma

**Figure 7:**
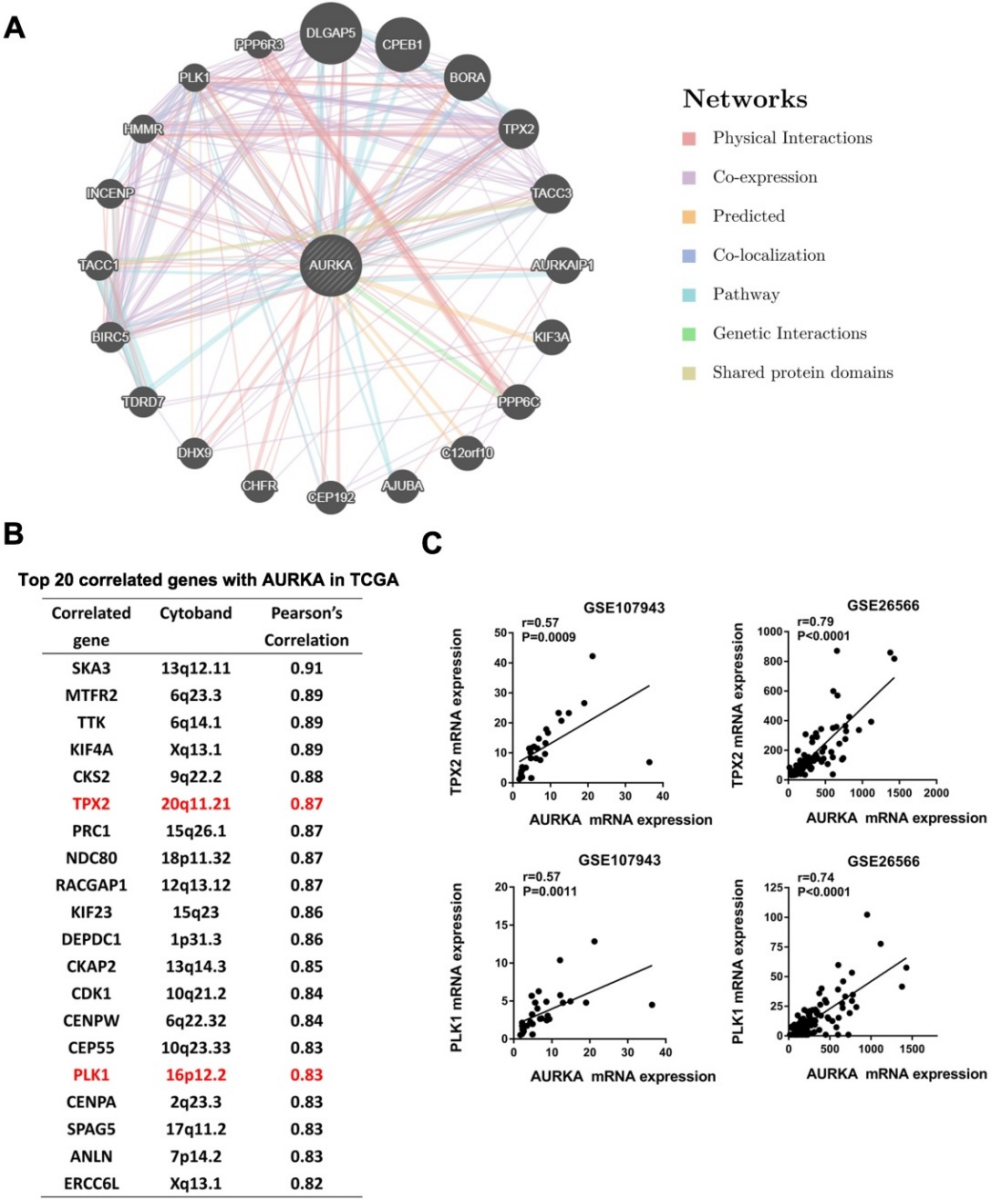
** AURKA correlated with TPX2 and PLK1. A)** Genetic interaction network of AURKA generated with the help of the GeneMANIA online tool. The top 20 genes were shown. Blue and pink lines denote pathways and physical interactions, respectively. **B)** Top 20 correlated genes with AURKA in the TCGA dataset of cholangiocarcinoma based on the Person's analysis. **C)** The expression level of AURKA showed a significant positive correlation with TPX2 and PLK1 expression in the dataset of GSE107943 and GSE26566. The r and P values were determined by Pearson correlation analysis.

To identify network genes that may collaborate with AURKA in promoting cholangiocarcinoma, we generated a Genetic interaction network of AURKA using the online tool GeneMANIA (www.genemania.org). Among the top 20 genes that may have an interaction with AURKA were TPX2 and PLK1 (Fig. 7A). These are two well-established co-factors of AURKA in various cell lineages but not been reported in cholangiocarcinoma. We sought to confirm the co-expression of TPX2, PLK1 and AURKA in cholangiocarcinoma datasets. Notably, both TPX2 and PLK1 were among the top 20 correlated genes with AURKA in the TCGA dataset of cholangiocarcinoma based on Person correlation analysis (Fig. 7B). Additionally, we verified a significantly high correlation of AURKA with TPX2 and PLK1 in the dataset of GSE107943 (r= 0.57 and 0.57 respectively) and GSE26566 (r=0.79 and 0.71 respectively) (Fig. 7C).

## Discussion

Despite cholangiocarcinoma being one of the most malignant cancers, the molecular processes that underlie its development are relatively understudied. Limited success has so far been achieved in respect of diagnostic and prognostic markers in cholangiocarcinoma. In addition, no targeted therapy has so far been approved against this neoplasm. To identify new putative drug targets, we compared gene expression profiles between cholangiocarcinoma tissues and normal bile ducts and identified global upregulation of mitotic spindle-associated genes. To our knowledge, this is the first report of a broad overexpression of mitotic spindle checkpoint components as a common characteristic of human cholangiocarcinoma.

AURKA play key roles in regulating mitotic entry, centrosome maturation and separation, and bipolar spindle assembly. AURKA has been reported to be overexpressed in various cancers and has been shown to be a promising target in cancer 16-23. However, no study specifically investigates the role and function of AURKA in cholangiocarcinoma. In this study, we found that AURKA was highly expressed in cholangiocarcinoma and high AURKA expression was a good predictor of poor prognosis in cholangiocarcinoma. The negative prognostic role of AURKA in cholangiocarcinoma was solid as it was validated in three different cholangiocarcinoma cohorts. AURKA expression was also highly correlated with expression of proliferation marker Ki-67. Hence, AURKA expression is associated with enhanced proliferation of tumor cells. Next, we evaluated the impact of the AURKA inhibitor on cholangiocarcinoma cells *in vitro*. Alisertib (MLN8237) is an orally available ATP-competitive, highly selective inhibitor of AURKA (40-fold selective for AURKA compared with AURKB) 24. Clinical trials testing Alisertib as a single agent or in combination with conventional chemotherapies are ongoing for the treatment of various malignancies 14, 25-33. Of the entire AURKA inhibitors, only Alisertib has proceeded to phase III evaluation 34. Therefore, we chose Alisertib for further studies. Application of Alisertib on cholangiocarcinoma cells inhibited cell growth and induced apoptosis *in vitro*. As a key regulator of cell cycle, AURKA regulates G2/M transition. Consistent with this, we found Alisertib arrested cells in G2/M phase. Additionally, we showed the *in vivo* capacity of Alisertib to arrest tumor growth in a mouse xenograft model of cholangiocarcinoma without obvious toxicity. Finally, AURKA depletion through siRNA achieved a similar effect with Alisertib, excluding the off target effect of Alisertib. Therefore, both *in vitro* and *in vivo* results support AURKA might be an effective therapeutic target in cholangiocarcinoma.

Our study was consistent with a recent high-throughput screening study which showed that Aurora kinase inhibitors were effective in all cholangiocarcinoma cell lines tested 35. AURKA inhibition is attractive in cholangiocarcinoma as it has been shown to interact with and control a variety of proteins that play key roles in cholangiocarcinoma pathogenesis such as STAT3 36 and Mcl-1^22^.

Although the AURKA currently represent the most accessible therapeutic target among the genes we identified, other members of the mitotic spindle checkpoint may warrant further study as potential prognostic markers of disease or alternate drug targets.

Our data demonstrate that AURKA was highly expressed and associated with poor prognosis in cholangiocarcinoma. Phenotypic changes post kinase inhibition or depletion indicates that AUKRA was involved in mediating cell proliferation, cell cycle progression, and apoptosis resistance. Furthermore, inhibition of AURKA using a highly specific small inhibitor Alisertib has proven here to be promising in targeting cholangiocarcinoma. Considering the lack of established targeted therapeutics against cholangiocarcinoma, we propose that AURKA may be a potential prognostic marker and effective therapeutic target for cholangiocarcinoma. In addition, we identified spindle checkpoint dysregulation as a common feature of human cholangiocarcinoma. We believe that the spindle assembly checkpoint in human cholangiocarcinoma warrants further investigation to explore the anticancer potential of drugs that target this pathway.

## Supplementary Material

Supplementary figure.

## Abbreviations

AURKA: Aurora kinase A
AURKB: Aurora kinase B
CCA: Cholangiocarcinoma
CCK-8: Cell counting kit-8
DFS: Disease free survival
OS: Overall survival
GEO: Gene Expression Omnibus
PLK1: Polo-like kinase 1
TCGA: The Cancer Genome Atlas
TPX2: Targeting protein for xenopus kinesin-like protein 2
