# Supplementary material for: Therapeutic Rationale to Target Highly Expressed Aurora kinase A Conferring Poor Prognosis in Cholangiocarcinoma
Source: J Cancer. 2020 Feb 3;11(8):2241–51. doi: 10.7150/jca.31989 (PMC7052919; doi:10.7150/jca.31989)

supplementary Figure. HuCCT1 cells were injected subcutaneously into the right flanks of athymic nude mice. When tumors reached a size of approximately150- 200 mm^3, mice were randomized to receive vehicle (n=6) or Alisertib (n=6) by oral gavage for 25 days. Pictures of nude mice and tumors were shown.

A B

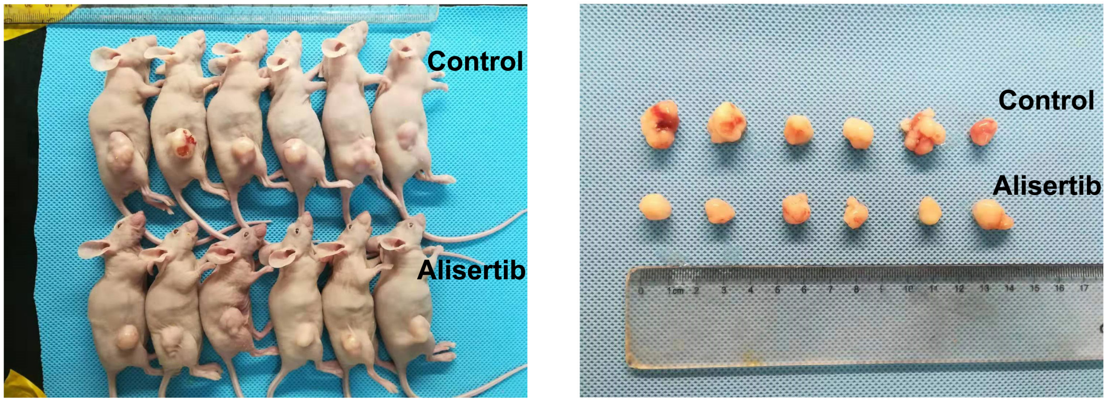

Supplement: Supplementary file 1 — Supplementary figure. [file jcav11p2241s1.pdf]
